# Empower People… With Money

**DOI:** 10.3389/ijph.2023.1605932

**Published:** 2023-07-03

**Authors:** Irina Bergenfeld

**Affiliations:** Hubert Department of Global Health, Emory University, Atlanta, GA, United States

**Keywords:** global health, development, empowerment, cash transfers, funding

**The IJPH series “Young Researcher Editorial” is a training project of the Swiss School of Public Health.**

In the global health world, the term “empowerment” has become as contentious as it is ubiquitous. We promote empowerment, measure it, and throw money at it. Despite over two decades of intense discourse around the term, empowerment is still difficult to define and even more challenging to operationalize. Some have concluded that we should entirely abandon the concept. Whatever our definition, one thing is clear: empowerment must begin with the assumption that people understand their own needs. But to build on this foundation, many global health practitioners must drastically re-evaluate the work we do and learn to trust that the presumed beneficiaries of our grant money are qualified to decide what to do with it.

I’d like to tell a story that exemplifies how and why the most well-intentioned efforts to empower others often fail to achieve their ends. I had the good fortune to work for project led by a well-known international NGO and an eminent university designed to reduce child marriage in south Asia. A lot of donor money had been invested in this project, which focused on changing social norms and engaging girls to become advocates within their communities. Some donor money was spent to fly me over, ostensibly to assist our very capable in-country partners with monitoring, but also to spend down our allocated travel budget. Although my presence was not necessary to the success of the project, I gained valuable personal and professional experience from this work.

The project did not fail entirely. Most participants reported enjoying the sessions and felt their experiences were positive. But the project did not reduce child marriage: our impact evaluation revealed that child marriage rates had already been quite low at baseline, possibly because the legal age of marriage (20 years) was being enforced. Moreover, the girls most likely to get married as teenagers were also least likely to take part in the program. These girls came from socioeconomically vulnerable homes and were already at a disadvantage when the program began. The impact evaluation confirmed the comments we had received from locals in interviews and focus groups: social norms in their communities had already shifted away from child marriage, but poor families still sometimes married their daughters off early because they needed the money. Our project focused on changing social norms but did not address the broader structural inequalities that shaped the lives of girls and their families.

This is an all too common story in the field of behavioral interventions, where cause and effect are rarely straightforward. Our theories of change often cannot account for the broader contexts in which they are tested. Within the sphere of public health, the role social determinants play in the health of both individuals and populations is increasingly recognized. So how can we, as public health professionals, empower people to reduce social and economic inequality? The answer is both obvious, and highly controversial. We must give direct financial assistance to those in need. As scientists and implementers, we can and should aim to a higher standard when we design and evaluate interventions that we believe will improve the lives of others. We spend donor and taxpayer money (sometimes in ways that do not benefit our intended beneficiaries, such as airfare for low-level project staff) to test whether our interventions work better than nothing (and we often find they do not), when simply giving people money and letting them decide how to spend may produce better results.

There is mounting evidence that cash transfer programs successfully improve a wide range of health outcomes, including child nutrition and healthcare usage [1–3]. Most criticisms of cash transfers are based on moralistic hand-wringing about dependency, qualms about encouraging laziness, and a persistent need to infantilize the poor [4], while evidence suggests that, if anything, cash transfers increase adult labor participation in low- and middle-income countries [1].

Of course, one could argue that funders will hesitate to contribute to more costly evaluation studies in which the counterfactual group receives cash transfers. But funders should be interested in finding out whether their money would be better spent by eliminating the intermediary (and those pesky indirect costs). Some organizations have begun to embrace this principle: for example, USAID already uses “cash-benchmarking” as a way to evaluate nutrition and youth job training programs against a cash transfer “standard of care” [5, 6]. Cash-transfer controls may well become an industry standard in global health; at the very least, the evidence should encourage scientists, implementers, and funders to challenge their assumptions about where global health money is most effectively spent.
